# Machine Learning–Driven Design of Sustainable Polymer Membranes: Integrated Prediction of Gas Permeability, Selectivity, and Biodegradability

**DOI:** 10.1021/acsomega.5c10251

**Published:** 2025-12-18

**Authors:** Haruki Ochiai, Kazukiyo Nagai, Hiromasa Kaneko

**Affiliations:** Department of Applied Chemistry, School of Science and Technology, 12939 Meiji University, 1-1-1 Higashi-Mita, Tama-ku, Kawasaki, Kanagawa 214-8571, Japan

## Abstract

Increasing CO_2_ emissions are causing environmental pollution worldwide, and there is a growing need for the development of efficient CO_2_ separation and recovery technologies. Many polymer materials, including polymer membranes, are difficult to decompose once released into the environment, leading to environmental pollution and adverse effects on ecosystems, and accordingly, biodegradable materials are required. In this study, we focused on the gas permeability and biodegradability of polymer materials and developed machine learning models to explore highly selective CO_2_ separation polymer membrane materials and predict their biodegradability. For gas permeability prediction, we used a data set of polymer membranes with standardized synthesis and film-forming conditions, confirming that highly accurate gas permeability coefficient predictions were possible even with a small number of polymer samples. Furthermore, we predicted the gas permeability coefficients and gas selectivity of 3219 polymer material candidates and identified approximately 500 high-performance polymers that exceed the Robeson upper bounds. Then, the polymers predicted to have biodegradability were also included. The present machine-learning framework enables us to propose computational candidates for CO_2_-separation membranes that are predicted to exhibit both high gas separation performance and biodegradability within the studied chemical space, providing hypothesis-generating guidance for future experimental studies.

## Graphical abstract

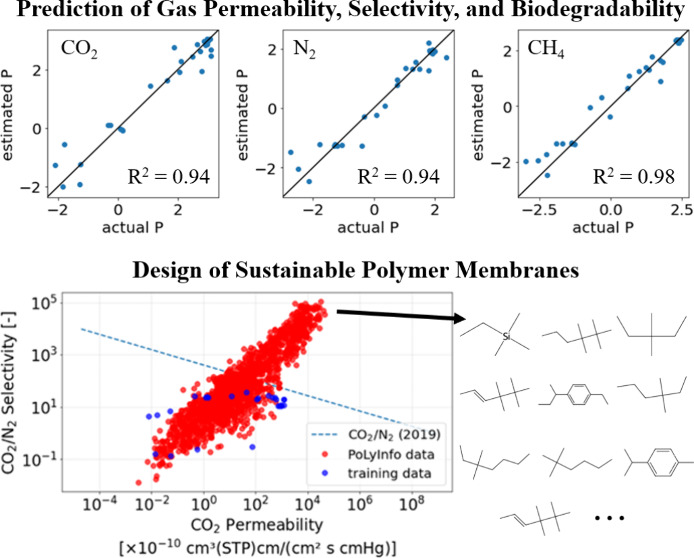

## 1 Introduction

Global environmental problems are becoming more serious as CO_2_ emissions increase, and it is required to develop technologies to efficiently separate and recover CO_2_ from major emission sources such as power and industrial plants. The membrane separation method, one of the CO_2_ separation and recovery technologies, uses organic material membranes such as polymers and inorganic material membranes such as zeolites that utilize the difference in gas permeation velocity.^1^ The separation performance of polymer membranes is evaluated based on two indexes: gas permeability and gas selectivity, and the development of materials with both the indexes is required. However, there is a trade-off between permeability and selectivity, and the pareto optimal solutions or the Robeson upper bounds^2–4^ have been updated several times since 1991. The design of membrane materials that exceed the bounds has become important in the development of membrane separation technology.

In recent years the field of polymer informatics has shifted markedly toward advanced deep-learning and graph-based representations. For example, Phan et al. demonstrated a multitask learning approach combining experimental and simulation data to predict permeability, diffusivity and solubility in polymers, thereby improving generalizability to novel chemistries.^5^ Gao et al. (2024) introduced self-supervised graph neural networks that incorporate polymer chain architecture, monomer stoichiometry and graph-level pretraining for improved property prediction in data-scarce domains.^6^ More recently, Huang et al. proposed a large-scale multimodal polymer representation framework that integrates multiple data modalities to enhance prediction and design of polymer materials.^7^

The performance of polymer membranes varies depending on synthesis and processing conditions, in addition to monomer structures. For example, even when polymers are synthesized based on the same raw materials, differences in polymerization method, heat treatment, presence or absence of cross-linking, and crystallinity affect gas permeability and selectivity. Therefore, we focused on a data set with uniform synthesis conditions to construct machine learning models predicting gas permeability and selectivity from monomer structures and establish design guidelines for polymer membrane materials, and thus, used a data set of the same research institute under unified experimental conditions for reliable evaluation of polymer membrane materials and design of new materials.

Many conventional polymer materials, including polymer membranes, are difficult to decompose once released into the environment, causing problems of environmental pollution and adverse effects on the ecosystem.^8^ It is required to develop environmentally friendly materials with biodegradability, which can be decomposed into water and CO_2_ by hydrolysis and microbial action. Although various organizations, including the organization for economic co-operation and development (OECD),^9^ have proposed standard test methods for evaluating biodegradability, a problem is that the evaluation varies from test to test and that the tests take a long time and costs.^10^ It is desirable to develop machine learning models that can predict biodegradability without any experiments.

The objective in this study is to explore novel CO_2_ separation polymer membrane materials with high gas permeability and selectivity and to evaluate their biodegradability. Machine learning models are constructed to predict gas permeability coefficients and to design polymer membrane materials that can exceed the Robeson upper bounds by inverse analysis of the models. When constructing the models, only experimental data from the same institution is used to unify the polymerization conditions. Then, to select the most environmentally friendly polymer membrane materials, we construct machine learning models to predict the biodegradability of the polymer membrane materials. Then, polymer membrane materials with high permeability, selectivity, and biodegradability can be designed.

Numerous studies have applied machine learning to predict gas transport properties of polymer membranes and accelerate the discovery of high-performance materials. For instance, Barnett et al. developed a topological fingerprint–based ML model trained on ∼ 700 experimental permeability records, enabling the in silico screening of over 11,000 homopolymers and the experimental validation of two candidates exceeding the Robeson upper bound.^11^ Xu et al. proposed an explainable graph machine learning approach, demonstrating predictive designs that outperform existing materials in multiple gas separation tasks.^12^ Phan et al. introduced a multitask learning framework that fuses high-fidelity experimental data with low-fidelity simulation data to predict permeability, diffusivity, and solubility simultaneously, achieving enhanced generalization across chemical space.^5^ Basdogan et al. integrated machine learning with a genetic algorithm to perform constrained inverse design, discovering promising polymer membranes for CO_2_ separations.^13^ A recent review by Li et al. provides a comprehensive overview of ML-assisted gas separation membrane development, covering the full workflow from data representation to candidate design.^14^

While these studies have advanced polymer informatics, most permeability prediction works rely on data sets collected under varying synthesis and evaluation conditions, introducing significant variability that can affect prediction reliability. Furthermore, although the use of RDKit descriptors and PoLyInfo data is common, such approaches often lack integration with other material performance metrics beyond permeability and selectivity.

In contrast, the present study uses permeability data measured entirely under uniform synthesis and evaluation conditions from a single institution, thereby reducing variability and improving model credibility. We extend the design objective beyond permeability and selectivity to also include biodegradability, enabling the identification of multifunctional polymer membranes that combine high CO_2_ separation performance with low environmental impact.

Biodegradability prediction for polymers has received less attention in prior literature and is typically treated as a standard binary classification task using existing data sets. However, these data sets differ substantially across international testing guidelines (e.g., OECD, EU), and small sample sizes in certain guidelines limit model accuracy. In this work, we address these issues by introducing a guideline-specific transfer learning (TL) and linear interpolation (LI) strategy, which significantly improves prediction accuracy in small data sets. To our knowledge, this is the first integration of permeability/selectivity prediction and guideline-specific biodegradability modeling in polymer informatics. Moreover, predictions are filtered through applicability domain (AD) evaluation to ensure chemical space similarity between training and candidate data sets, thereby enhancing reliability.

In this work, we develop a reliability-aware dual-screening workflow that combines independently trained models for gas permeability/selectivity and biodegradability under their respective ADs, and apply it to explore computational candidates for sustainable CO_2_-separation membranes.

In this study, we (i) construct machine-learning models to predict gas permeability of polymers using repeating-unit descriptors and a uniform-condition permeability data set, (ii) screen database polymers for CO_2_/N_2_ and CO_2_/CH_4_ separation performance relative to Robeson-type bounds, (iii) develop guideline-specific biodegradability classifiers and apply them under AD constraints, and (iv) combine these components into a reliability-aware dual-screening workflow to generate computational candidates for sustainable polymer membranes.

## 2 Methods

### 2.1 Molecular Descriptors for Monomer Structures

The structural information used for descriptor calculation was derived from the repeating unit of each polymer, represented in SMILES notation. These repeating units are not isolated small molecules but structural fragments corresponding to polymer backbones or copolymer segments. Although the visualized repeating units may appear short (e.g., small aliphatic or siloxane fragments), they serve as representative chemical motifs used to compute molecular descriptors that capture polymer-level chemical characteristics.

For copolymers, the descriptors of each repeating unit were calculated separately and then averaged according to the copolymer composition ratio, thereby approximating the overall chemical environment of the polymer. Consequently, all models developed in this study predict polymer-level properties such as gas permeability and biodegradability based on these repeating-unit representations.

RDKit^15^ was used to calculate molecular descriptors for monomer structures of polymer materials. The RDKit descriptors consist of zero to two dimensions. The descriptors for each dimension represent compositional descriptors in zero dimension, fragment counts in one dimension, and topological descriptors in two dimension.

### 2.2 Experimental Data

Experimental data measured under low pressure conditions (supply pressure of 1 atm) near room temperature, where pressure dependence and changes over time did not need to be considered, were used. The permeability coefficient, *P* [cm^3^ (STP) cm/(cm^2^ s cmHg)], was calculated with the experimental data from the following equation:

$$P=\frac{dp}{dt}\times \frac{273V}{760T}\times \frac{1}{A}\times \frac{1}{p}\times l$$ 1

where d*p /* d*t* is the steady-state rate of pressure increase, *v* [cm^3^] is the downstream volume, *T* [K] is the temperature, *A* [cm^2^] is the area of the film, *p* [cmHg] is the upstream pressure, *l* [cm] is the film thickness, the upstream pressure was maintained at about 1 atm, and the downstream side was vacuum. All permeability data were measured on at least three times to ensure reproducibility.

The diffusion coefficient *D* [cm^2^/ s] is calculated from the time lag θ before reaching steady state as follows:

$$D=\frac{l^{2}}{6\theta }$$ 2

The solubility coefficient *S* [cm^3^ (STP)/(cm^3^ cmHg)] is calculated with *P* and *D* as follows:

$$S=\frac{P}{D}$$ 3

### 2.3 Data Interpolation

There exist missing values and values can be discontinuous for time in a data set. The missing or discontinuous values affect the predictive accuracy of machine learning models and require appropriate interpolation, and thus, should be appropriately handled. LI is a method connecting data points by a straight line and uses the values on the line to estimate new data points, which assumes a constant rate of change between adjacent data points and interpolates missing or skipped values.

Iterative Gaussian mixture regression (iGMR)^16^ is one of the nonlinear methods used for missing value interpolation and uses Gaussian mixture regression (GMR)^17,18^ to estimate missing values. In a sample containing missing values, GMR can be used to estimate missing values from the data points of the other variables.

### 2.4 Transfer Learning

When the number of samples in a target data set is small, the model has a risk to overfit to the data set. If there is a support data set with a large number of samples that has similar characteristics to the target data set, more accurate predictions can be achieved by combining these data sets to construct a machine learning model. This technique, known as TL, is particularly effective when the target and support data have the same target variable Y. Frustratingly easy domain adaptation^19^ was used in this study.

### 2.5 Applicability Domain of the Model

By inputting values of explanatory variables X into a constructed machine learning model, it is possible to predict Y value or class even for samples for which actual Y value or class is unknown. However, it is difficult to determine whether the predicted value or class is reliable because the model does not have predictive ability when samples that differ significantly from the training data are input. Therefore, it is important to set the AD of the model^20^ to evaluate the reliability of the model for an input sample. In this study. k-nearest neighbors (kNN), which can calculate an index of data density, was used to set the AD. The predictions of Y are reliable when an input sample of X is within AD.

## 3 Results and Discussion

### 3.1 Construction of Machine Learning Models Predicting Gas Permeability Coefficients

Polymer membrane materials were collected from literature at the same laboratory.^21–31^ For model construction, the regression methods given as follows were used in this study:

- OLS: Ordinary least-squares regression^32^
- PLS: Partial least-squares regression^33^
- RR: Ridge regression^34^
- LASSO: Least absolute shrinkage and selection operation^35^
- EN: Elastic net^36^
- LSVR: Linear support vector regression with linear kernel^37^
- NLSVR: Nonlinear support vector regression with Gaussian kernel^37^
- DT: Decision tree^38^
- RF: Random forests^39^
- GPR: Gaussian process regression with nonlinear kernels^40^
- GBDT: Gradient boosting decision tree^41^
- XGB: Extreme gradient boosting^42^
- LGBM: Light gradient boosting machine^43^

RDKit descriptors and physical properties such as *D*, glass transition temperature (Tg), and density were used as explanatory variables X. Tg and density were not specified in some papers, and accordingly, the missing values were interpolated using iGMR. Because copolymers were included in a data set, weighted average of RDKit descriptors whose weights were composition ratios was used as X. As a preprocess of X, X with more than 95% of the same values were removed. Y was a log transformed *P*. The predictive ability of models was evaluated using the determinant coefficient R^2^, mean absolute error (MAE), and root mean squared error (RMSE) after double cross validation (DCV),^44^ which was performed using the leave-one-out method due to the small number of samples to evaluate the predictive ability of models. In the outer loop of DCV, the model was repeatedly trained on a subset of the data set and evaluated on experimentally measured polymer samples that were not included in the training process. Additionally, the performance metrics (R^2^, MAE, RMSE) reported in this work are calculated after integrating over the outer folds of DCV procedure, and thus reflect the typical generalization performance to unseen experimental polymers within the studied data set. Therefore, the obtained R^2^, MAE, and RMSE values represent the ability of models to predict new experimental polymer data within the studied chemical space, providing a reliable internal validation of predictive performance.

Tables 1 and 2 show the three methods with the largest R^2^ after DCV for each gas for the case where X is only RDKit descriptors and the one where X is RDKit descriptors, D, Tg, and density, respectively. Plots of measured vs predicted Y corresponding to the results with the largest R^2^ after DCV are shown in Figures 1 and 2, for Tables 1 and 2, respectively. Since *D* of H_2_ cannot be measured, the results for H_2_ are not shown in Table 2 and Figure 2. While the predictions were accurate for all gases shown in Table 1 and Figure 1, the prediction errors for the polymers with the largest measured *P* values for CO_2_ and O_2_ were large and the predictions were much low than the measured values. In DCV, the sample with the largest measured Y value was predicted with the model constructed using the other samples with the small values of *P*, and from the DCV results, the model could not be constructed to explain the sample with the largest measured Y value. Then, the prediction accuracy of the models improved by adding physical properties to X as shown in Table 2 and Figure 2, compared to the results of only RDKit descriptors. The importance of physical properties such as *D*, Tg, and density in the prediction of *P* was confirmed.

**Table 1:** Prediction Results of the Three Methods with Large *R* ^2^ after DCV for Each Gas when X Was Only RDKit Descriptors

gas	method	*R* ^2^	MAE	RMSE
H_2_	GPR 1	0.911	0.253	0.378
	GPR 9	0.911	0.255	0.379
	Ridge	0.903	0.261	0.395
N_2_	GPR 1	0.924	0.323	0.431
	GPR 9	0.921	0.327	0.439
	GBDT	0.916	0.319	0.453
CO_2_	LASSO	0.822	0.500	0.750
	GPR 4	0.803	0.423	0.789
	GPR 6	0.803	0.423	0.789
O_2_	GBDT	0.752	0.395	0.763
	GPR 8	0.748	0.398	0.769
	GPR 6	0.748	0.398	0.769
CH_4_	GBDT	0.950	0.294	0.405
	DT	0.950	0.323	0.405
	GPR 9	0.947	0.295	0.417

**Table 2:** Prediction Results of the Three Methods with Large *R* ^2^ after DCV for Each Gas when X Was RDKit Descriptors, *D*, Tg, and Density

gas	method	*R* ^2^	MAE	RMSE
N_2_	GBDT	0.937	0.262	0.392
	GPR 8	0.929	0.324	0.416
	GPR 2	0.929	0.325	0.417
CO_2_	GPR 6	0.939	0.303	0.440
	GPR 2	0.939	0.304	0.441
	GPR 0	0.939	0.304	0.441
O_2_	LSVR	0.933	0.274	0.395
	GPR 4	0.921	0.317	0.429
	GPR 6	0.920	0.318	0.430
CH_4_	GBDT	0.976	0.215	0.264
	EN	0.956	0.250	0.359
	LASSO	0.952	0.255	0.376

**Figure 1:**
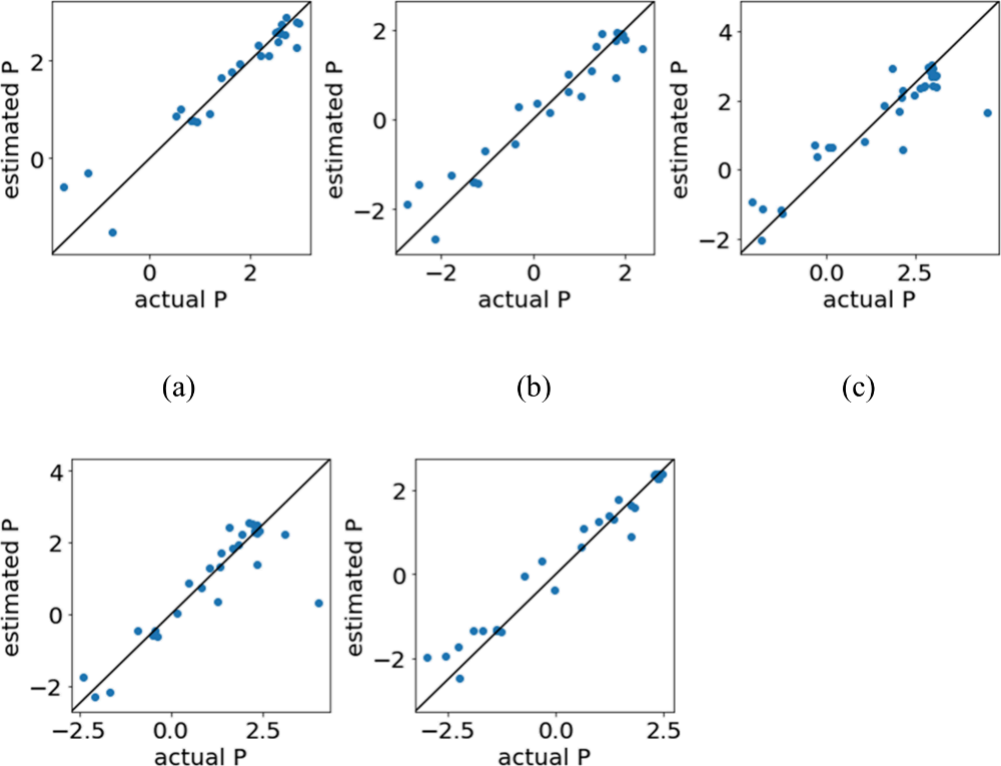
Plots of measured *P* vs predicted *P* for the method with the largest *R* ^2^ after DCV for each gas when only RDKit descriptors were used as X. (a) H_2_, GPR1, (b) N_2_, GPR1, (c) CO_2_, LASSO, (d) O_2_, GBDT, and (e) CH_4_, GBDT.

**Figure 2:**
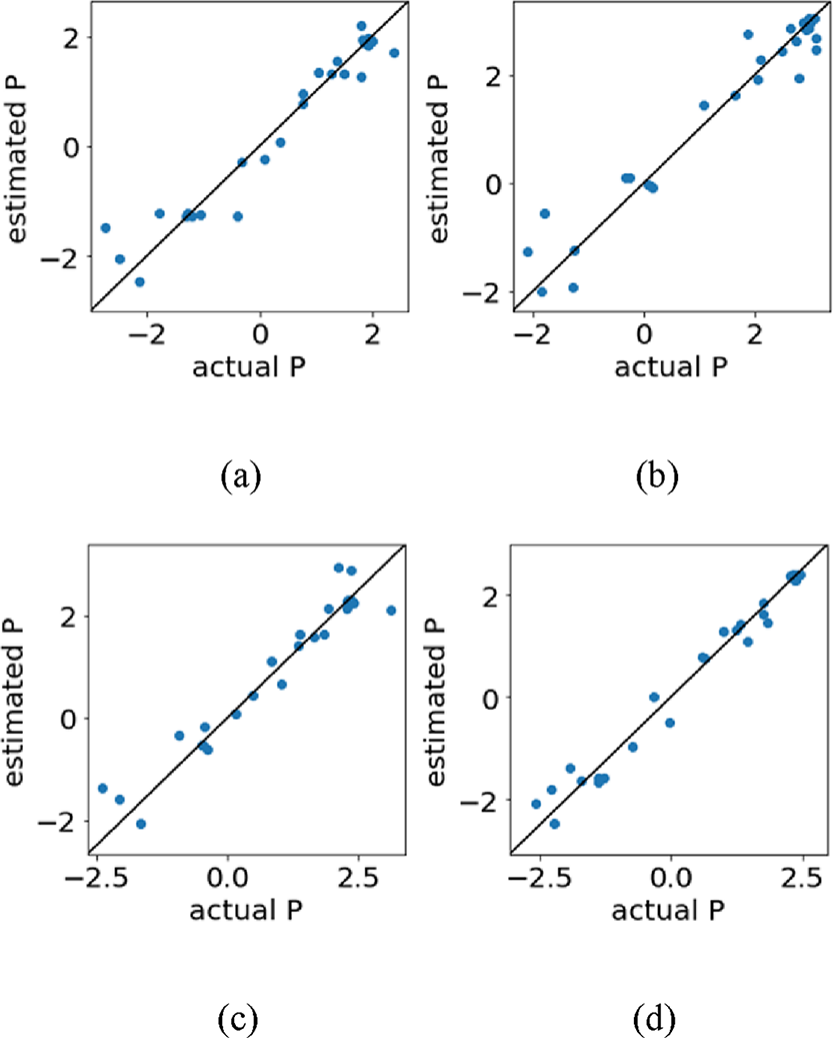
Plot of measured *P* vs predicted *P* for the method with the largest *R* ^2^ after DCV for each gas when RDKit descriptors, *D*, density, and Tg were used as X. (a) N_2_, GBDT, (b) CO_2_, GPR6, (c) O_2_, LSVR, and (d) CH_4_, GBDT.

For all gases, the inclusion of physical properties (D, Tg, and density), some of which were interpolated for missing entries, led to consistently higher R^2^ and lower prediction errors compared with models based solely on structural descriptors, indicating their usefulness within the present data set. At the same time, the use of interpolated values may partially inflate correlations, which should be kept in mind when interpreting the results.

Because the permeability data set is relatively small and chemically narrow, the generalizability of the models is inherently limited, and some variability of performance across DCV folds is expected. The averaged metrics reported in this study should therefore be interpreted as indicative values rather than precise global estimates, and external validation using independent data sets from other laboratories is an important subject for future work.

### 3.2 Design of New CO_2_ Separation Membrane Polymer Materials

The models constructed in the previous section were used to predict *P* of other polymer materials with unknown permeability, aiming to find polymers with high CO_2_ permeability and high CO_2_/N_2_ and CO_2_/CH_4_ selectivity for CO_2_ separation membranes. For a data set of polymer materials to be predicted, we used 3219 polymers collected from PoLyInfo.^45^ For polymers with unknown experimental permeability coefficients, only structural information was used to predict the gas permeability coefficients, and thus, the models using only RDKit descriptors as X were used. The regression analysis methods used were the optimal method for each gas, which were shown in Figure 1.

Figure 3 shows the predicted CO_2_ permeability coefficients, and the predicted CO_2_/N_2_ and CO_2_/CH_4_ selectivity, which were calculated from eqs 1) and 2) with predicted permeability coefficients of CO_2_, N_2_ and CH_4_ for the polymers in AD, and they are compared to the Robeson upper bounds. In Figure 3, the CO_2_/N_2_ and CO_2_/CH_4_ separation performances of database polymers are compared with the Robeson upper bounds. Here, the positions of the data points are determined from the predicted permeability coefficients and selectivities obtained by the machine learning models, whereas the Robeson bounds themselves are taken directly from the literature as established empirical trends and are not rederived in this work. The many polymer materials were found to exceed the Robeson upper bounds. Of the predicted permeability coefficients and selectivity, 590 polymers exceeded the Robeson upper bounds for CO_2_/N_2_ separation and 492 polymers for CO_2_/CH_4_ separation. Our machine learning models proposed new candidates of polymer membrane materials with high CO_2_ permeability and high CO_2_/N_2_ and CO_2_/CH_4_ selectivity.

**Figure 3:**
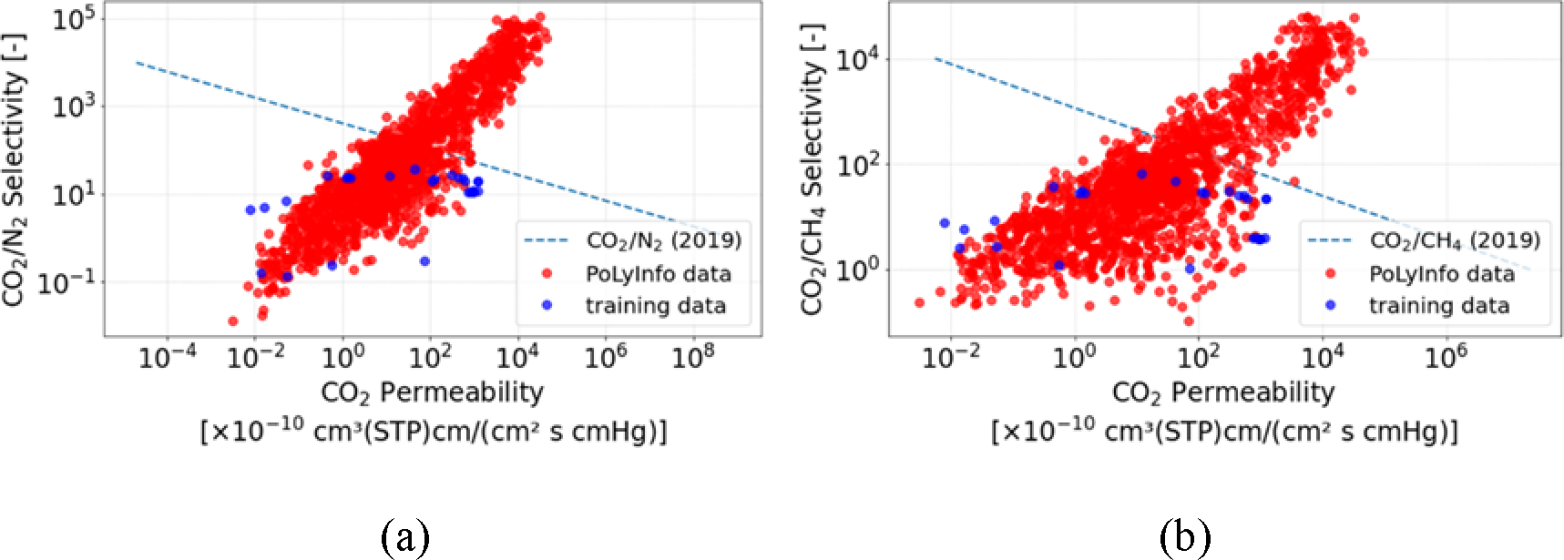
Distribution of polymers for predicted CO_2_ permeability and selectivity of (a) CO_2_/N_2_ and (b) CO_2_/CH_4_. Red points indicate data collected from PoLyInfo, blue points indicate training data, and dot lines indicate the Robeson upper bounds in 2019.

The polymer candidates with low predicted permeability shown in Figure 3 would be applied to packaging materials because they exhibit high barrier properties. Since high-barrier materials contribute to quality retention and extended shelf life in food and pharmaceutical packaging, our proposed method was useful not only in the design of membrane materials with high separation performance, but also in the design of high-barrier membrane materials.

In the PoLyInfo screening, only polymers lying within AD of the corresponding models were considered for further analysis. As a result, polymers outside the AD—although potentially interesting—were not evaluated in this study, and exploring such regions of chemical space remains an important direction for future work.

### 3.3 Biodegradability Prediction Model Construction

Data sets of experimental biodegradability tests were collected from the literature.^10^ The guidelines and the numbers of samples are shown in Table 3. The original data sets showed biodegradability [%], and then, we classified them into two classes: readily biodegradable (RB) if the material was determined to be RB in the environment and not readily biodegradable (NRB) if the material was determined not to biodegrade. The threshold was set at 70% for OECD guideline 301 A, OECD guideline 301 E, OECD guideline 302 B, and EU Method C.4-A, and 60% for other guidelines.

**Table 3:** Number of Samples per Guideline

guideline		# of samples
OECD	301 A	357
	301 B	2153
	301 C	1576
	301 D	1409
	301 E	165
	301 F	2091
	302 B	206
	302 C	248
	310	260
EU	C.4-A	113
	C.4-C	46
	C.4-D	175
	C.4-E	122

In each data set, although there exist a lot of data for measuring biodegradability at the general test period of 28 days, there exist especially little data for shorter periods of time. We used LI to expand the data set. LI was performed for compounds that contained three or more measurements at different number of days. Because biodegradability increases with time, compounds with experimental values exceeding the RB criterion before 28 days are expected to exceed the criterion after the last date of elapsed days as well. Therefore, in addition to the LI, even when the maximum number of days of experimental values was less than 28 days, if the threshold of RB was exceeded at that maximum number of elapsed days, the data were expanded to include biodegradability up to 28 days as RB. These allowed the data to be extended even in the range where experimental values were not available.

Although biodegradation is an irreversible phenomenon that cannot be reversed once it has progressed, experimental results varied, and there were cases where the degree of biodegradation increased or decreased even for the same compound. In particular, data for the same compound and under the same guidelines (measurement conditions) that showed an increase or decrease to the extent that the presence or absence of biodegradability changed were deleted because the reliability of the data was questionable. Then, some guidelines have more than 2000 samples, while others have as few as 100 samples. When a biodegradability prediction model is constructed for each guideline, a model with a small number of samples cannot provide adequate prediction performance. Although simple way would be to integrate the data sets, the biodegradability in the data set varies with each guideline in terms of measurement environment and method. Therefore, we integrated the data sets of the guidelines with FEDA as TL to predict the biodegradability of each guideline.

RDKit descriptors and elapsed days were used as X and two classes: RB and NRB were used as Y. The classification methods used for model construction are listed as follows:

- LR: Logistic regression^46^
- LDA: Linear discriminant analysis^47^
- NB: Naive Bayes^48^
- kNN
- NLSVM: Nonlinear support vector machine with Gaussian kernel^37^
- DT
- RF
- GBDT
- XGB
- LGBM

DCV was used to evaluate the predictive ability of models, with the number of inner fold was five and the number of outer fold was 10 for OECD Guideline 301 B, C, D, and F due to the large number of samples, and leave-compound-out for the other guidelines.

Four patterns were used for model construction: simple per-guideline (normal), LI, TL, and a combination of TL and LI. The evaluation indexes with the best prediction performance in DCV for each guideline are shown in Table 4. For OECD Guideline 301E, 302B, and 310, LI improved the prediction accuracy of models. These guidelines have a small sample size of approximately 200, and data expansion would be useful. On the other hand, OECD Guideline 302C and EU Method C.4-D showed a decrease in the prediction accuracy of models with LI. In particular, EU Method C.4-D has only 14 samples of RB data, and the excessive increase in NRB data with LI would result in an imbalance of samples of two classes. In addition, TL + LI was the most accurate in OECD Guideline 302C. The usefulness of data expansion in small numbers of data, but it was important to check the balance of samples of two classes after interpolation to prevent a decrease in prediction accuracy of models.

**Table 4:** Evaluation Indices in the Biodegradability Prediction Model in DCV

		method	accuracy	recall	precision	F-score
OECD Guideline 301 A	normal	LGB	0.720	0.808	0.712	0.757
	LI	LGB	0.695	0.767	0.698	0.731
	TL	LGB	0.675	0.777	0.673	0.721
	TL + LI	LGB	0.717	0.793	0.715	0.752
OECD Guideline 301 B	normal	XGB	0.786	0.656	0.711	0.682
	LI	LGB	0.793	0.632	0.738	0.681
	TL	XGB	0.798	0.663	0.735	0.697
	TL + LI	XGB	0.782	0.629	0.714	0.669
OECD Guideline 301 C	normal	LGB	0.841	0.725	0.764	0.744
	LI	XGB	0.841	0.735	0.759	0.746
	TL	LGB	0.843	0.733	0.763	0.747
	TL + LI	LGB	0.838	0.762	0.737	0.750
OECD Guideline 301 D	normal	XGB	0.776	0.570	0.682	0.621
	LI	LGB	0.746	0.478	0.642	0.548
	TL	XGB	0.777	0.595	0.675	0.632
	TL + LI	LGB	0.781	0.581	0.689	0.631
OECD Guideline 301 E	normal	NB	0.636	0.778	0.516	0.620
	LI	NB	0.697	0.825	0.571	0.675
	TL	NB	0.630	0.873	0.509	0.643
	TL + LI	NB	0.648	0.746	0.528	0.618
OECD Guideline 301 F	normal	XGB	0.827	0.730	0.760	0.745
	LI	XGB	0.806	0.714	0.721	0.718
	TL	LGB	0.821	0.718	0.752	0.735
	TL + LI	LGB	0.812	0.696	0.742	0.718
OECD Guideline 302 B	normal	XGB	0.709	0.613	0.597	0.605
	LI	XGB	0.704	0.560	0.600	0.579
	TL	XGB	0.714	0.627	0.603	0.614
	TL + LI	LR	0.665	0.680	0.531	0.596
OECD Guideline 302 C	normal	NB	0.484	0.775	0.329	0.462
	LI	NB	0.548	0.704	0.355	0.472
	TL	LGB	0.710	0.437	0.492	0.463
	TL + LI	LGB	0.750	0.338	0.615	0.436
OECD Guideline 310	normal	XGB	0.723	0.667	0.679	0.673
	LI	XGB	0.731	0.667	0.692	0.679
	TL	XGB	0.742	0.694	0.700	0.697
	TL + LI	XGB	0.708	0.739	0.636	0.683
EU Method C.4-A	normal	LR	0.779	0.776	0.731	0.752
	LI	XGB	0.796	0.837	0.732	0.781
	TL	LGB	0.788	0.837	0.719	0.774
	TL + LI	LGB	0.823	0.898	0.746	0.815
EU Method C.4-C	normal	LR	0.935	0.917	0.846	0.880
	LI	LDA	0.913	0.917	0.786	0.846
	TL	LDA	0.870	0.917	0.688	0.786
	TL + LI	LGB	0.870	0.833	0.714	0.769
EU Method C.4-D	normal	NB	0.931	0.500	0.583	0.538
	LI	XGB	0.926	0.286	0.571	0.381
	TL	LR	0.937	0.429	0.667	0.522
	TL + LI	NB	0.943	0.500	0.700	0.583
EU Method C.4-E	normal	LGB	0.869	0.529	0.529	0.529
	LI	LR	0.852	0.529	0.474	0.500
	TL	NB	0.820	0.588	0.400	0.476
	TL + LI	XGB	0.852	0.471	0.471	0.471

### 3.4 Evaluation of biodegradability

The biodegradability of new polymer materials candidates predicted in Section 3.2 was evaluated using the model “Normal” of OECD Guideline 301F, which was developed in Section 3.3. OECD Guideline 301F is widely used to predict the environmental fate of compounds and is applicable even when compounds have low solubility in water, making it a test that can evaluate biodegradability for a wide variety of compounds.^49^ Therefore, it would be appropriate for use with compounds whose biodegradability is unknown.

The OECD 301F “Normal” model was selected for screening because its data set covered a relatively broad range of chemical structures with a more balanced distribution of readily RB and NRB outcomes compared with other guidelines. Nevertheless, its finite F-score implies that both false positives and false negatives may occur. Consequently, the RB predictions for the screened polymers should be regarded as model-based suggestions that require experimental confirmation.

The biodegradability was predicted for 28 days, a test period. Figures 4 and 5 show the top 10 candidates with the highest CO_2_ permeability and CO_2_/N_2_ selectivity among the CO_2_/N_2_ separation membrane materials that were predicted to be RB for 28 days, with 107 of the 590 CO_2_/N_2_ samples exceeding the Robeson upper bounds, respectively, and Figures 6 and 7 show the top 10 candidates with the highest CO_2_ permeability and CO_2_/CH_4_ selectivity among the CO_2_/CH_4_ separation membrane materials that were predicted to be RB for 28 days, with 99 of the 492 CO_2_/CH_4_ samples exceeding the Robeson upper bounds, respectively. Many of the structures with one or two aromatic rings were predicted to be biodegradable. Structures with multiple elements and complex structures were not observed in many of the structures. Biodegradable molecules are generally known to have aliphatic structures with ether bonds. However, structures containing aromatic rings were selected as a result of biodegradability prediction. The biodegradability model suggests that some simple aromatic motifs may still be compatible with RB behavior within the data sets considered, indicating that conventional empirical rules may not fully describe the observed patterns.

**Figure 4:**
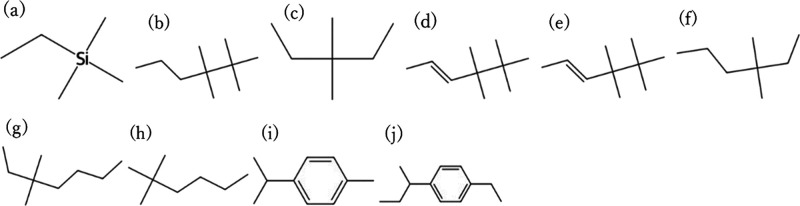
Top 10 monomer structures with the highest CO_2_ permeability among the CO_2_/N_2_ separation membrane candidates that were predicted to be RB. (a) CC­[Si]­(C)­(C)­C, (b) CCCC­(C)­(C)­C­(C)­(C)­C, (c) CCC­(C)­(C)­CC, (d) CC=CC­(C)­(C)­C­(C)­(C)­C, (e) C/C=C/C­(C)­(C)­C­(C)­(C)­C, (f) CCCC­(C)­(C)­CC, (g) CCCCC­(C)­(C)­CC, (h) CCCCC­(C)­(C)­C, (i) Cc1ccc­(C­(C)­C)­cc1, and (j) CCc1ccc­(C­(C)­CC)­cc1. These are included in the PoLyInfo database. The structures shown are repeating units representing the polymer backbones or copolymer segments and should be interpreted as design motifs rather than complete polymer chains.

**Figure 5:**
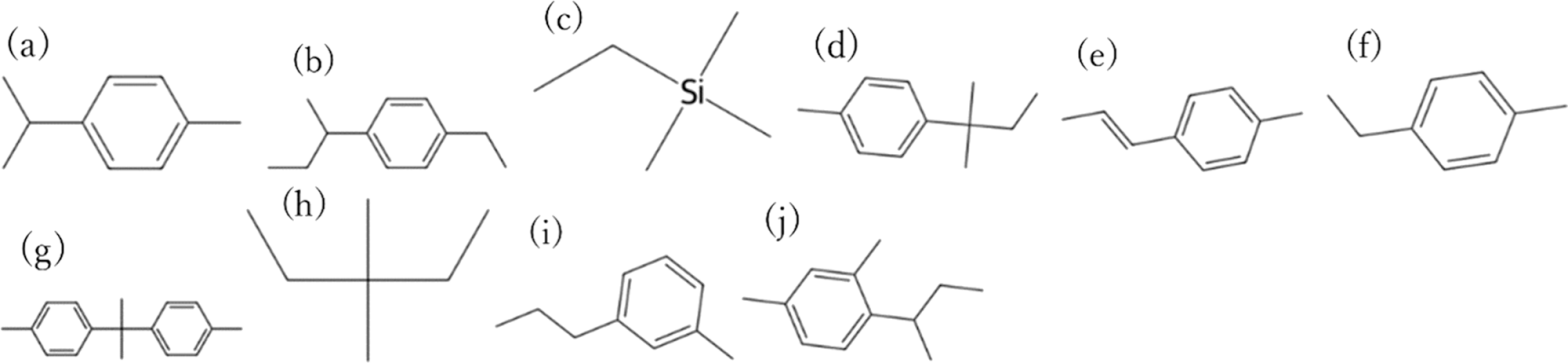
Top 10 molecular structures with the highest CO_2_/N_2_ selectivity among the CO_2_/N_2_ separation membrane candidates that were predicted to be RB. (a) Cc1ccc­(C­(C)­C)­cc1, (b) CCc1ccc­(C­(C)­CC)­cc1, (c) CC­[Si]­(C)­(C)­C, (d) CCC­(C)­(C)­c1ccc­(C)­cc1, (e) CC=Cc1ccc­(C)­cc1, (f) CCc1ccc­(C)­cc1, (g) Cc1ccc­(C­(C)­(C)­c2ccc­(C)­cc2)­cc1, (h) CCC­(C)­(C)­CC, (i) CCCc1cccc­(C)­c1, and (j) CCC­(C)­c1ccc­(C)­cc1C. These are included in the PoLyInfo database. The structures shown are repeating units representing the polymer backbones or copolymer segments and should be interpreted as design motifs rather than complete polymer chains.

**Figure 6:**
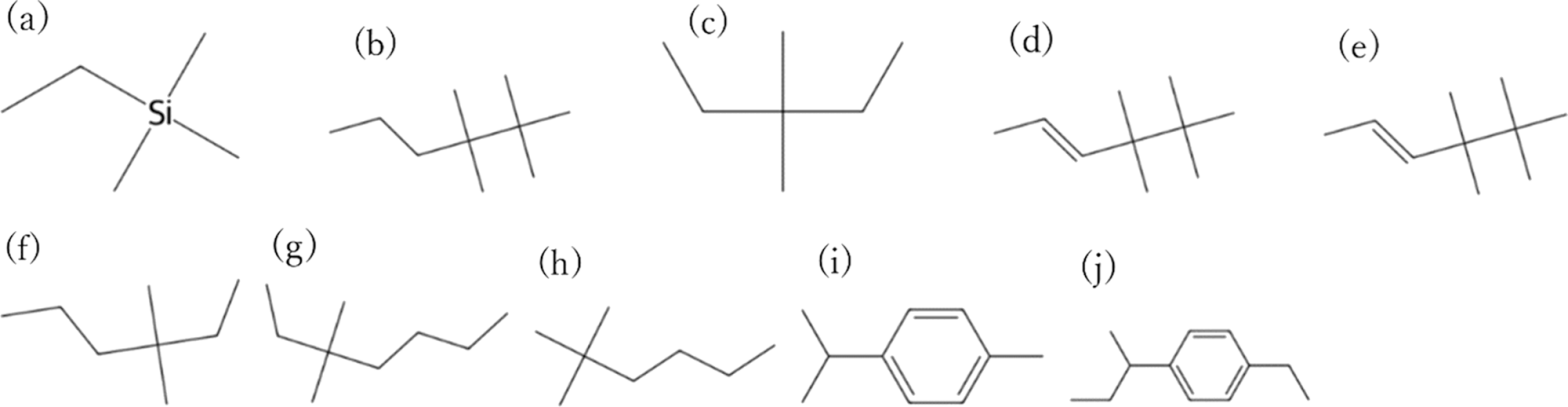
Top 10 molecular structures with the highest CO_2_ permeability among the CO_2_/CH_4_ separation membrane candidates that were predicted to be RB. (a) CC­[Si]­(C)­(C)­C, (b) CCCC­(C)­(C)­C­(C)­(C)­C, (c) CCC­(C)­(C)­CC1, (d) CC=CC­(C)­(C)­C­(C)­(C)­C, (e) C/C = C/C­(C)­(C)­C­(C)­(C)­C, (f) CCCC­(C)­(C)­CC, (g) CCCCC­(C)­(C)­CC, (h) CCCCC­(C)­(C)­C, (i) Cc1ccc­(C­(C)­C)­cc1, and (j) CCc1ccc­(C­(C)­CC)­cc1. These are included in the PoLyInfo database. The structures shown are repeating units representing the polymer backbones or copolymer segments and should be interpreted as design motifs rather than complete polymer chains.

**Figure 7:**
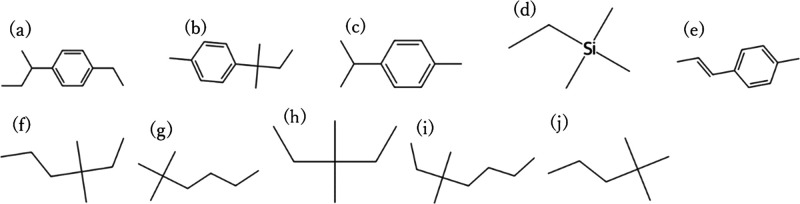
Top 10 molecular structures with the highest CO_2_/CH_4_ selectivity among the CO_2_/ CH_4_ separation membrane candidates that were predicted to be RB. (a) CCc1ccc­(C­(C)­CC)­cc1, (b) CCC­(C)­(C)­c1ccc­(C)­cc1, (c) Cc1ccc­(C­(C)­C)­cc1, (d) CC­[Si]­(C)­(C)­C, (e) CC=Cc1ccc­(C)­cc1, (f) CCCC­(C)­(C)­CC, (g) CCCCC­(C)­(C)­C, (h) CCC­(C)­(C)­CC, (i) CCCCC­(C)­(C)­CC, and (j) CCCC­(C)­(C)­C. These are included in the PoLyInfo database. The structures shown are repeating units representing the polymer backbones or copolymer segments and should be interpreted as design motifs rather than complete polymer chains.

The biodegradability classifiers developed in this study are based on guideline-specific data sets and descriptors derived from repeating units, and thus primarily reflect the behavior of relatively small molecules tested under standardized OECD/EU conditions. In real environments, polymer biodegradation is additionally influenced by factors such as polymer microstructure, cross-linking, additives, and transformation products, which are not explicitly represented in the present models. Therefore, the predictions obtained here should be considered as screening-level hypotheses rather than definitive assessments of environmental fate.

As a qualitative case study, we examined several of the top-ranked candidates in Figures 4–7 in terms of their resemblance to known membrane chemistries. Many of the high-permeability motifs resemble flexible aliphatic or siloxane-like backbones, which are consistent with materials known to exhibit high gas transport, whereas other candidates contain more rigid aromatic segments that may provide higher selectivity. At the same time, very simple branched alkanes or siloxane-rich motifs may suffer from challenges such as low mechanical robustness or difficulty in achieving high molecular weight. These considerations highlight that the present results should be interpreted as chemically plausible design motifs rather than immediately deployable membrane materials, and that experimental evaluation is essential for assessing their real feasibility.

It should also be emphasized that the motifs presented in Figures 4–7 are repeating unit representations and do not directly account for the full macromolecular architecture, molecular weight, or processing history of real membrane materials. In particular, very simple branched alkanes or siloxane-like motifs may face challenges such as poor mechanical robustness or difficulty in achieving high molecular weight and film integrity. Therefore, the present screening results should be viewed as motif-level design suggestions, which need to be embedded into chemically and mechanically feasible polymer architectures and validated experimentally.

It is important to note that the present framework is a sequential screening scheme rather than a formal multiobjective optimization. The permeability/selectivity and biodegradability models are trained independently and then combined at the decision level under AD constraints. In future work, this screening pipeline could be extended to genuine multiobjective design, for example by using Pareto-front or Bayesian multiobjective optimization over permeability, selectivity, and biodegradability (and, potentially, additional feasibility descriptors such as glass transition temperature and mechanical robustness).

## 4 Conclusions

We focused on the properties of gas permeability and biodegradability in polymer materials to search for new highly selective CO_2_ separation polymer membrane materials and to predict the biodegradability of organic compounds. For permeability prediction, gas permeability prediction models were constructed using a data set of materials whose gas permeability coefficients were measured. In the prediction of gas permeability of polymer membranes in DCV, the prediction could be performed with high accuracy even for a small number of samples using a data set with uniform conditions of membrane synthesis and evaluation for all gases. In the biodegradability prediction, biodegradability classification models were constructed using data sets of compounds for which biodegradability was tested based on a total of 13 OECD and EU guidelines. Although there were many samples with measured biodegradability at 28 days, which was a common test period, the data set was expanded using LI and TL, especially to address the problem that there existed few samples for shorter periods. For guidelines such as OECD Guideline 302 B, which had a small number of samples, LI and TL greatly improved the prediction accuracy of models, and the results showed that expansion of the data set for a small number of samples was useful. The constructed models were used to predict the gas permeability coefficients of new candidates of polymer materials with unknown gas permeability coefficients. We calculated the gas permeability coefficients and gas selectivity for 3219 material candidates and identified 590 candidates for CO_2_/N_2_ separation membranes and 492 for CO_2_/CH_4_ separation membranes that exceeded the Robeson upper bounds, which were considered the conventional limits. Furthermore, some of the polymers were predicted to be biodegradable, suggesting candidates for multifunctional polymer membrane materials with high performance and low environmental impact.

In addition to permeability and biodegradability, other polymer properties such as glass transition temperature (Tg), thermal stability, and mechanical strength play essential roles in determining the practical feasibility of polymer membranes. Although Tg and density were partially considered in this study as explanatory variables, future work will focus on integrating additional descriptors that capture thermal and mechanical robustness as well as processability. Such an expansion of the model framework will enable a more comprehensive and realistic evaluation of polymer performance for sustainable CO_2_ separation applications.

Although the present study did not include experimental synthesis or permeability measurements of newly proposed polymer candidates, the predictive reliability of the models was confirmed using experimentally measured but unseen data through the DCV framework. This ensures that the predictive performance reflects realistic generalization to new experimental polymers. In future work, we plan to synthesize and experimentally evaluate several of the promising polymers identified in this study.

One of the main objectives of this study was to overcome the conventional trade-offs among permeability, selectivity, and biodegradability in polymer membrane design. By developing separate machine learning models for each property and applying them within their respective ADs, we could identify candidate polymers predicted to achieve high separation performance and biodegradability simultaneously. Although experimental synthesis and measurement remain necessary to confirm these predictions, the present approach demonstrates the potential of data-driven molecular exploration to discover new polymers that may surpass traditional trade-offs in material performance.

In the present study, polymer structures were represented by descriptors derived from repeating units, and the models did not explicitly incorporate macromolecular features such as chain rigidity, free volume distribution, or segmental dynamics. Although the use of a uniform-condition experimental data set helps to absorb some of these effects, extending the framework to include polymer-level structural and morphological information (for example through 3D or graph-based descriptors and simulated free-volume characteristics) will be an important direction for future work.

In this study, the permeability/selectivity and biodegradability models were trained independently and then combined at the workflow level in a reliability-aware dual-screening pipeline under their respective ADs. Thus, the present framework should be regarded as a sequential screening scheme, rather than a fully unified multiobjective optimization. Extending this workflow to formal multiobjective design – for example, Pareto or Bayesian multiobjective optimization over permeability, selectivity, biodegradability, and additional feasibility descriptors – is an important direction for future work.

It should be emphasized that the polymers identified in this work are computationally screened candidates, and their practical performance as membrane materials must be confirmed by future synthesis and gas-permeation experiments. The present study therefore provides a hypothesis-generating framework for sustainable membrane discovery rather than a definitive identification of commercially ready materials. We hope that the proposed method promotes the development of polymeric membrane materials.

## Data Availability

We used the data set in the literature,^16–26^ and the data that support the findings of this study are available in ref 40. The molecular structures used and proposed in this study were indicated as SMILES in the captions in Figures 4–7. The permeability data used in this study were collected from previously published works by the same laboratory, as listed in the references. The PoLyInfo database is publicly accessible. Other processed data sets and the code used for model construction and screening are available from the corresponding author upon reasonable request.
